# Impact of Urban Morphology and Climate on Heating Energy Consumption of Buildings in Severe Cold Regions

**DOI:** 10.3390/ijerph17228354

**Published:** 2020-11-11

**Authors:** Shiyi Song, Hong Leng, Han Xu, Ran Guo, Yan Zhao

**Affiliations:** 1 School of Architecture, Harbin Institute of Technology, Harbin 150006, China; 17b934034@stu.hit.edu.cn (R.G.); 18S134198@stu.hit.edu.cn (Y.Z.); 2 Key Laboratory of Cold Region Urban and Rural Human Settlement Environment Science and Technology, Ministry of Industry and Information Technology, Harbin 150006, China; 3 Department of Educational Psychology, Faculty of Education, University of Macau, Macau 999078, China; honeytsui94@gmail.com

**Keywords:** building energy efficiency, energy use intensity, stepwise regression, spatial cluster

## Abstract

This study aims to acquire a better understanding of the quantitative relationship between environmental impact factors and heating energy consumption of buildings in severe cold regions. We analyze the effects of five urban morphological parameters (building density, aspect ratio, building height, floor area ratio, and shape factor) and three climatic parameters (temperature, wind speed, and relative humidity) on the heating energy use intensity (EUI) of commercial and residential buildings in a severe cold region. We develop regression models using empirical data to quantitatively evaluate the impact of each parameter. A stepwise approach is used to ensure that all the independent variables are significant and to eliminate the effects of multicollinearity. Finally, a spatial cluster analysis is performed to identify the distribution characteristics of heating EUI. The results indicate that the building height, shape factor, temperature, and wind speed have a significant impact on heating EUI, and their effects vary with the type of building. The cluster analysis indicated that the areas in the north, east, and along the river exhibited high heating EUI. The findings obtained herein can be used to evaluate building energy efficiency for urban planners and heating companies and departments based on the surrounding environmental conditions.

## 1. Introduction

According to the International Energy Agency (IEA), the building and construction sector should be the primary focus of attempts to reduce greenhouse gas emissions as it accounted for the largest share of global final energy use (36%) and energy-related CO_2_ emissions (39%) in 2018 [1]. Heating energy demand is responsible for a significant amount of the total energy consumption of a building. Reducing heating energy consumption is considered as an important step in meeting energy saving targets and preventing climate change, especially in areas with severe cold [2]. In China, the average carbon emission intensity from residential buildings that require heating is around four times higher than those that do not require heating [3]. Although the heating energy use intensity (EUI) reduced dramatically to 114 kWh/m^2^ in 2015, nearly 60% of that in 2001, total heating energy consumption is still growing as the total building area for heating has tripled [4]. Consequently, in the coming decades, energy-efficiency planning strategies in severe cold regions must involve adaptation measures to reduce the heating demand of buildings.

**Figure 1:**
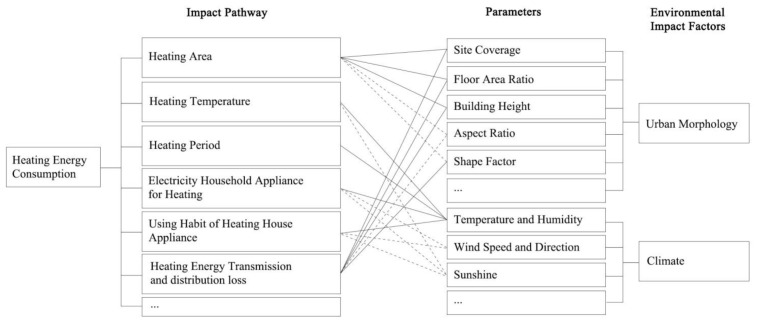
Environmental impact factors of heating energy consumption of buildings (solid lines represent direct impact; dashed lines represent indirect impact) [12].

Besides the physical condition of a building, such as the building type [5], construction [6], and HVAC (Heating, Ventilation and Air Conditioning) system [7], and its energy consumption patterns (ECP) [8,9], such as energy-use behavior and lifestyles, numerous studies have observed that the surrounding environment is critical in influencing the building heating energy consumption (Figure 1) [10,11,12]. Urban morphology and climatic conditions have been identified as key environmental factors that affect the overall heating demand in cities [13,14,15]. Considering urban morphology, Mouzourides et al. [13] performed a multiscale analysis on a validated dataset of urban energy demands to investigate the effects of building height and building density on heating and cooling demand. Ahn and Sohn [16] also confirmed that building height is a potential driver of the energy demand of a building. Martins et al. [14] observed that aspect ratio and building height accounted for almost 50% of the overall impact on heating demand. Furthermore, floor area ratio and shape factor are also energy-related urban morphological parameters [11,17]. The impact of climatic conditions has been widely discussed as well. Xie et al. [18] analyzed the impact of air temperature, relative humidity, global solar radiation, and wind speed on building heating demand, based on data collected from field measurements. These four parameters are the main energy-related climate variables in most climate models that investigate the potential impacts of climate change on building heating and cooling energy demands, and they have been applied in several studies in Switzerland [19], Australia [20], the U.S. [21,22], and Austria [23].

Various methods have been proposed to study building energy demand and can be categorized into the white-box, grey-box, and black-box methods [24,25,26,27,28,29]. White-box methods require the detailed physical information of buildings to ensure higher accuracy [26]. Grey-box methods are an improvement over white-box methods and use a combination of physical information and historical data to construct a building energy simulation model [25,26]. Black-box methods were developed to overcome the defect of computational inefficiency and are essentially based on historical data instead of physical information. The advantages of black-box methods have steadily increased due to continuous development and calibration, and they have become quite prominent in large-scale building energy consumption research. Several popular methods such as multiple linear regression (MLR), the bin method (BM), support vector regression (SVR), artificial neural networks (ANN), and Gaussian process regression (GPR) are black-box methods. MLR can successfully predict building energy consumption [25] and can offer even higher accuracy than ANN with proper modification and optimization and carefully selected parameters [26,30]. Ciulla and D’Amico [24] developed a multiple regression model to determine building energy demand and used the Pearson coefficient to select suitable variables, ensuring high reliability and usability. Amiri, Mottahedi, and Asadi [31] combined multiple regression analysis with building energy simulation software. They used stepwise regression to identify the most effective parameters and used the R statistical analysis software to develop a set of linear regression equations. In this study, we develop an MLR model to quantify the relationship between impact factors and heating EUI.

This study aims to quantitatively explore the impact of urban environmental factors—urban morphological factors and climatic factors—on the heating energy use intensity of buildings in severe cold regions. The multiple linear regression approach was applied based on carefully selected empirical data of over 600 buildings, of six different building types, located across the main urban area of Harbin, China. The stepwise regression method ensured that the study only focused on the variables that have the highest impact on heating EUI. In addition, the distribution of heating EUI in the spatial dimension was also analyzed, by using cluster analysis to identify the distribution characteristics.

The rest of this article consists of four sections—the materials and methods, results, discussion, and conclusions. The materials and methods section includes a detailed description of the method of data selection and capture, the statistical analysis and verification process, and the cluster analysis approach. The impact and the final multiple regression model of the urban morphology and climatic conditions on each building type are explored in the subsequent section, as well as the main findings compared with existing studies, limitations, and the potential for future research.

## 2. Materials and Methods

### 2.1. Energy Consumption Data

**Figure 2:**
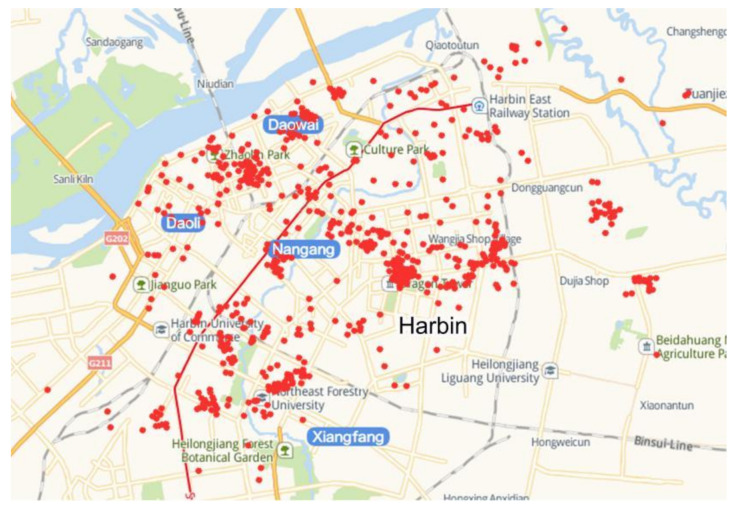
The location of selected buildings.

The building energy consumption database of this study consists of the measured heating energy consumption data and physical building information of 609 civil buildings, located across four administrative regions in the main urban district of Harbin, in 2017 (Figure 2). A part of the database was obtained from the Energy Monitoring System for National Large-Scale Public Buildings, and the rest was submitted by the heating company. Most cities in severe cold regions of China use central heating systems in winter which take hot water as the main heat source. The heating company is the main department responsible for adjusting the heat supply in the central heating system to the building according to the external temperature and heating standards [32]. For example, in Harbin, the heating company needs to supply enough heating energy to residential buildings to ensure that the indoor temperature is not lower than 20 ℃.

To standardize the comparison, the heating energy consumption was converted into energy use intensity, which is defined as the energy used per unit floor area, expressed in kWh/m^2^. To ensure the accuracy of the model and avoid excessive interference, data-cleaning is essential. Buildings with missing data, duplicate samples, and outliers were discarded. The outliers were identified by PauTa criteria. We first checked whether the data conform to the normal distribution. The sig. value of the test of normal was 0.06 (Table 1), indicating that the data were normally distributed. The mean value of heating EUI was 168.93 kWh/m^2^ and the standard deviation was 35.66 kWh/m^2^. Therefore, according to the PauTa criteria, heating EUI values higher than 275.90 kWh/m^2^ or lower than 61.96 kWh/m^2^ were regarded as outliers. The building age was limited to 30 years. This ensures that the heating system and construction methods of a given type of building are reasonably similar. Finally, 609 buildings, including 242 residential buildings and 367 commercial buildings, were selected. The commercial buildings were divided into 5 categories based on their operational characteristics—48 hotels, 50 retail buildings, 35 hospitals, 107 educational buildings, and 127 offices. The categories also limit the energy use schedule of each building. All selected buildings used central heating system in winter. The value of parameters of selected buildings were presented in Table 2.

**Figure 3:**
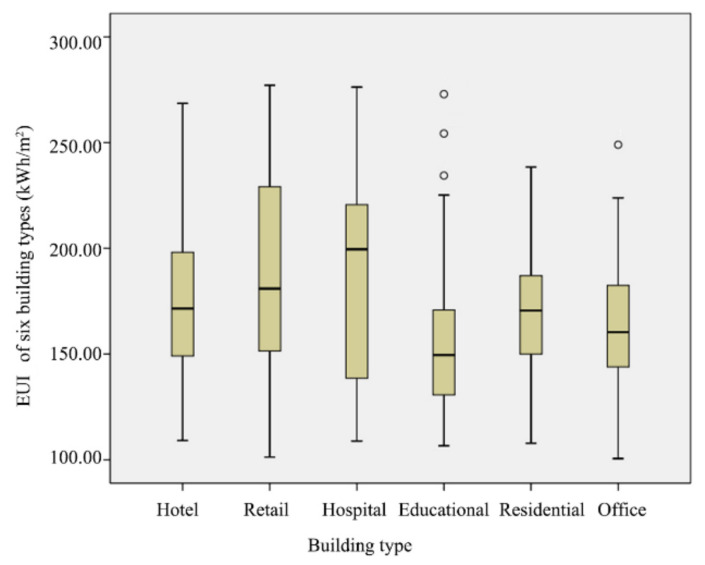
Box plot of heating EUI of different building types.

Figure 3 presents annual heating EUI of each building type. The median value of each type differs significantly, which indicates that heating EUI varies across building types. As expected, the highest median heating EUI is that of hospitals, possibly because of their constant medical needs. The lowest median heating EUI is that of educational buildings. A possible explanation is that some educational buildings are vacant for over a month during the winter and thus have an extremely low heating demand. Notably, despite the large sample size, the heating EUI of residential buildings is around 160 kWh/m^2^, indicating that there is a negligible difference in the heating demand between the different types of residential buildings, such as multi-storey, mid-rise, and high-rise buildings.

To validate the method, we used 85% of the available data to determine the regression model equations, while the remaining 15% was used to evaluate and test the reliability achieved by each model.

### 2.2. Environmental Impact Parameters

#### 2.2.1. Urban Morphological Parameters

Although the quantification of spatial parameters is one of the most important and widely used methodologies to describe urban morphology, the spatial indicators used in research and urban planning are not uniform [33,34]. Based on the purpose and objective of a study, the definition and calculation of an indicator can vary significantly. The spatial indicators used in this study are obtained from previous studies [17,35,36] and are limited by the amount of data that are captured. Therefore, the parameters chosen in this study are by no means the only input variables suitable for this purpose, and investigations with alternate parameters are encouraged.

##### Building Density (BD)

**Figure 4:**
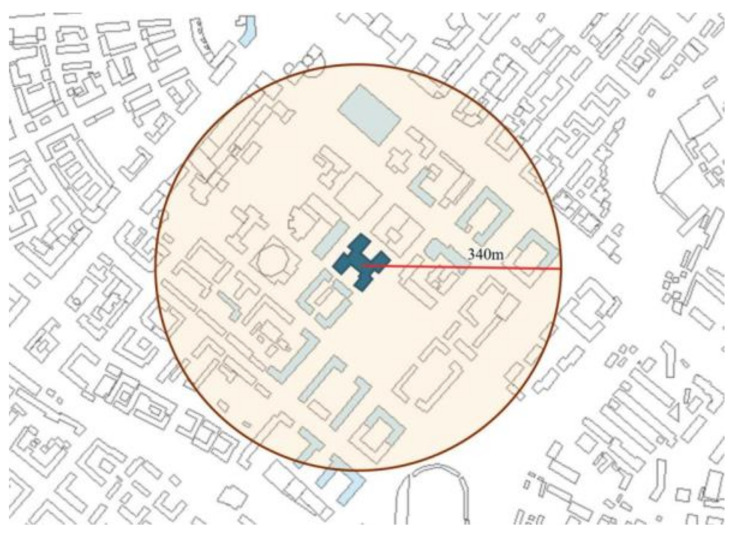
Neighborhood size set for building density calculation.

The main urban area is composed of buildings, and the building density of the surrounding neighborhoods significantly affects the natural ventilation of the area and the shadows between buildings [13,37,38]. The wind and shadows created by surroundings play a significant role in affecting the convective heat transfer coefficients at the exterior surfaces of a building [39], which in turn affects the heating load of the building [40]. Detailed research shows that the building density of the neighborhood has a strong impact on the energy consumption of residential and office buildings [38]. The method chosen to study the building density of the neighborhoods of each selected building is to use the geographic information system (GIS) data captured from Baidu Maps and calibrated through on-site surveys. Previous studies have shown that for the case of the downtown building, the effective radius of the surrounding environment is between 340 and 420 m [10]. In order to achieve the highest accuracy, we set the radius of environment as 340 m for building density calculation (Figure 4).

##### Floor Area Ratio (FAR)

Floor area ratio is another density-related parameter selected in this study. FAR impacts the solar potential of buildings by increasing shading by neighboring buildings [41,42]. The surrounding neighborhood size was defined with 340 m as the radius.

##### Aspect Ratio (AR)

The ambient temperature in the street canyon depends on the wind speed in the canyon. Aspect ratio was introduced by Oke [43] and defined as the ratio of building height to the width of the distance between buildings. These two elements significantly affect the distribution of wind speed in the street canyon and therefore affect the convective heat transfer from the surface of the building [10,44]. Previous studies have confirmed the effect of AR on building heating energy consumption [14].

##### Building Height (BH)

The height of the building may affect natural ventilation by restricting air flow within the urban canyon. However, despite reducing heat loss, deep street canyons created by a cluster of high-rise buildings also reduce solar gain, which results in the increase in the heating demand in the cold season [16,40].

##### Shape Factor (SF)

Shape factor reflects the ratio of the external surface area of a building in contact with the outdoor atmosphere and the volume enclosed by it. It is directly related to the energy saving of the building and mainly affects the solar radiation (heat and light) interactions [17]. It can be used to evaluate the total heat loss [40,42].

#### 2.2.2. Climatic Parameters

The climate data were collected from the National Meteorological Data Center and are based on the data measured by the Harbin National Basic Weather Station. The wind speed (WSP), temperature (TEMP), and relative humidity (RH) were defined as the key climatic parameters. The climatic data are differentiated by month, from January to April, and from October to December, in 2017.

##### Temperature

The temperature outside the building is widely recognized as a factor that has an important impact on building energy consumption [45]. Temperature not only affects the energy performance of the building itself, but it also affects the energy use behavior of the occupants inside the building.

##### Wind Speed

The air flow on the surface of the building will affect the convective heat transfer on the surface of the building, which shows great impact on the heat transfer, surface temperature, and cooling rate of the building shell [45].

##### Relative Humidity

Relative humidity has been considered in several studies as one of the parameters affecting the cooling and heating load of buildings [45,46]. It has been proven to be an important contributor to the urban heat island effect, which in turn affects building energy consumption [46,47].

### 2.3. Statistical Analysis Framework

**Figure 5:**
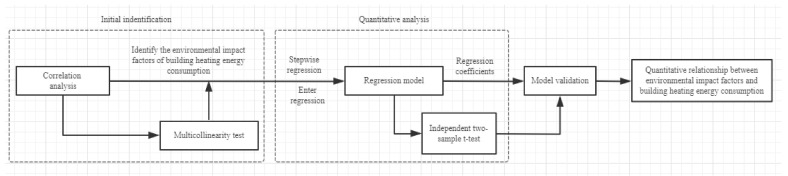
Statistical analysis framework.

The statistical analysis framework used in this study is shown in Figure 5.

#### 2.3.1. Correlation Analysis

To ensure an accurate statistical analysis, we designed a standard experimental procedure. Based on the purpose of the study, independent variables can have different levels of importance, and the number of variables can have a significant impact on the regression analysis. To verify the importance of the design variables and eliminate unimportant factors, a Pearson correlation analysis was conducted. Since none of the factors were considered to be the deciding factors of building heating demand, those with a correlation significance of 0.01 (2-tailed) or 0.05 (2-tailed) were all considered in the subsequent verification process.

#### 2.3.2. Multicollinearity Test

The correlation analysis can also be used to determine the degree of collinearity between independent variables. Multicollinearity within a set of independent variables can cause several problems in determining the significance of individual independent variables in the regression model [48]. Therefore, multicollinearity must be tested before the regression analysis is performed, so that the model can be suitably adjusted. The correlation coefficient and the variance inflation factor (VIF) are used as the test indicator. VIF is the ratio of the variance of the estimator of the regression coefficient to the variance when assuming a non-linear correlation between independent variables (Equation (1)). If the correlation coefficient is above 0.8 or VIF is above 5 [49], multicollinearity may exist between the variables and they may need to be removed from the model. The results of the multicollinearity test are used not only to select variables but also to determine which type of regression model is required.

(1) $$VIF=\frac{1}{1-R^{2}}$$

where $R_{i}^{2}$ is the coefficient of determination of the regression model of the independent variable *i* to other independent variables.

#### 2.3.3. Stepwise Regression

Stepwise regression is a step-by-step iterative construction of a regression model that involves the automatic selection of independent variables [50,51] and is widely used to identify the relationships between variables. It is also recognized as one of the methods used to solve the problem of multicollinearity of independent variables. Stepwise regression aims to determine the independent variables that have a significant influence on the dependent variable through a series of tests (F-tests, *t*-tests). In this study, we selected the forward selection stepwise regression, which tests each variable as it is added to the model and retains the variables that are most statistically significant until the results are optimal. The probability of F was set to 0.05 for entry and 0.10 for removal.

#### 2.3.4. Independent Two-Sample T-Test

To examine whether the independent variable in the regression model is correlated to the dependent variable, an independent two-sample *t*-test was performed and calculated as shown in (Equation (2)).

(2) $$t=\frac{\bar{X_{1}}-\bar{X_{2}}}{\sqrt{\frac{\left(n_{1}-1\right)S_{1}^{2}+\left(n_{2}-1\right)S_{2}^{2}}{n_{1}+n_{2}-2}\left(\frac{1}{n_{1}}+\frac{1}{n_{2}}\right)}}$$

where $\bar{X}$ is the average value of each sample set, *S*^2^ is the variance of each sample set, and *n* is the number of records in each sample set.

#### 2.3.5. Model Validation

An important step between model construction and model application is to test the rationality and applicability of the model. Usually, the determination coefficient (R^2^) is used as a measure of the predictability of a model. In this study, besides R^2^, we also conducted other validation approaches to monitor the stability of the model parameter estimates and whether the model could be used in a range outside the sample observations. We refer to the method of [24] to conduct a preliminary analysis of the distribution of standardized residuals and express the residuals through the residual graph. If the standardized residuals are all within the (−2, 2) interval, the experimental data can be considered reliable. In addition, four evaluation criteria are used to deeply analyze model errors, which are the mean absolute error (MAE) (Equation (3)), the mean absolute percentage error (MAPE) (Equation (4)), the mean square error (MSE) (Equation (5)), and the root mean square error (RMSE) (Equation (6)).

(3) $$MAE=\frac{1}{n}\overset{n}{\underset{i=1}{\sum }}\left|x_{i}-y_{i}\right|$$

(4) $$MAPE=100\cdot \frac{1}{n}\overset{n}{\underset{i=1}{\sum }}\frac{\left|xi-y_{i}\right|}{xi}$$

(5) $$MSE=\frac{1}{n}\overset{n}{\underset{i=1}{\sum }}{\left(xi-yi\right)}^{2}$$

(6) $$RMSE=\sqrt{\frac{1}{n}\overset{n}{\underset{i=1}{\sum }}{\left(xi-yi\right)}^{2}}$$

where *xi* is the *i*-th expected output value, *yi* is the *i*-th predicted value, *n* is the number of records in each sample set.

### 2.4. Cluster Analysis

To examine the spatial autocorrelation of the dependent variable, which is inherent to geo-referenced data, Moran’s *I* is used as the indicator. First, the Global Moran’s *I* is tested to describe the spatial distribution of the heating EUI in the entire area and to discover the spatial differences caused by the spatial correlation (Equations (7) and (8)). The value of Global Moran’s *I* ranges from −1 to 1, where a value of 0 implies no autocorrelation [52,53,54].

(7) $$I=\frac{n}{S_{0}}\frac{\sum_{i=1}^{n}\sum_{j=1}^{n}w_{i,j}\left(x_{i}-\bar{X}\right)\left(x_{j}-\bar{X}\right)}{\sum_{i=1}^{n}{\left(x_{i}-\bar{X}\right)}^{2}}$$

(8) $$S_{0}=\overset{n}{\underset{i=1}{\sum }}\overset{n}{\underset{j=1}{\sum }}wi,j$$

where *I* is the Global Moran’s *I* value for a feature i, x_i_ is an attribute of the feature *i*, $\bar{X}$ is the mean of the corresponding attribute, *w_i,j_* is the spatial weight between the features *i* and *j*, *S*_0_ is the aggregation of all spatial weights, and *n* equates to the total number of features.

Subsequently, a local indicators of spatial association (LISA) cluster map is created to augment the significant location with an indication of the type of spatial association, based on the location of the value and its spatial lag in the Moran scatter plot. Generally, five categories are represented—high-high, low-low, low-high, high-low, and not significant [52,54].

- “High-high” describes an area of high value surrounded by areas of high values.
- “Low-low” describes an area of low value surrounded by areas of low values.
- “Low-high” describes an area of low value surrounded by areas of high values.
- “High-low” describes an area of high value surrounded by areas of low values.
- “Not significant” describes an area whose *p*-value of local Moran’s *I* > 0.05.

In this study, the calculations of Moran’s I are conducted on the GeoDa software (GeoDa Center for Geospatial Analysis, University of Chicago, Chicago, U.S.). Each grid measures 340 × 340 m. The heating EUI value of each grid is calculated as the average value of the buildings in the grid. The distance weight is set by the K-nearest neighbors algorithm of four.

## 3. Results

### 3.1. Result of Initial Identification

The significance and direction of the impact of the variables can be identified from Table 3. The correlation results indicate that the impact of the urban morphological variables vary across building types. AR and BH have significant effects on the heating EUI of all building types except hospitals, whose heating EUI shows almost no response to variations in urban morphology. Residential buildings appear to be easily affected by all five urban factors and are the only building type that is significantly affected by FAR. These effects are exhibited in different ways. Increasing the AR and BH can decrease the heating EUI of hotels, educational buildings, residential buildings, and offices, while increasing BD, FAR, and SF leads to an increase in the heating EUI. As expected, higher AR and BH, or a lower street width, leads to a narrow construction distance, which increases the heat stored between buildings and reduces the heating energy demand. In contrast, a higher BD increases the length of the shadows cast by the buildings and leads to a reduction in the direct and diffused incoming solar energy. This reduction effect is considerably stronger than the increase in heat storage caused by higher density.

All three climate variables show significant correlation with the heating EUI of all the building types. The impacts of WSP and TEMP are negative, which implies that an increase in these variables may reduce the heating demand of the building. In contrast, the impact of RH is positive.

The initial correlation analysis can only reveal the relationships between the independent variables and the dependent variable. Strong linear correlations, which can significantly reduce the stability and accuracy of the regression model, may exist between the independent variables. Therefore, the multicollinearity between the variables must be tested. Table 4, Table 5 and Table 6 show the correlations between the independent variables. As expected, significant correlations (Pearson correlation > 0.8 or VIF > 5) exist between AR and BH for all building types except hospitals. This is true for the climatic variables as well. In other words, there is multicollinearity between AR and BH, as well as between the climate variables. Notably, when only two independent variables are involved, the VIF can be below 5—for instance, in educational and office buildings. Subsequently, the correlation coefficient is investigated to determine the degree of collinearity.

### 3.2. Result of Regression Models

#### 3.2.1. Urban Morphological Parameters

Stepwise regressions were performed on each building type, based on the impact of each variable identified by the correlation analysis. As shown in Table 7, the effects of the urban morphological variables vary across building types.

In the building types that show multicollinearity between AR and BH, AR was discarded during the regression analysis. This implies that BH has a higher impact on heating EUI than AR. In addition, BD was excluded in hotels and residential buildings. Although the heating EUI of residential buildings appears to be sensitive to all five urban morphological variables, FAR and SF have the highest impact and make the largest contribution to the R-squared value of the regression model. The R-squared values of the five regression models range from 0.074 to 0.272. As the urban morphological factors only constitute some of the impact factors of building heat demand and are not the deciding factors, the interpretive ability of the models is acceptable. All samples have passed the *t*-test, which means that the results of regression are repeatable.

As shown in Table 3, BH has a direct effect on the heating demand of four types of buildings. However, its impact on the heating EUI of residential buildings is not significant and it is removed from the regression model. The coefficients of BH in three types of buildings—hotels, educational buildings, and offices—are negative, which indicates that the higher the BH, the lower the heating EUI of building. The large variations in BH values with the type of building are due to the differences in building design and building energy use behavior, which can also be defined by the functions of the building and the daily schedule of the energy consumption of the occupants. The regression analysis revealed that when both BH and AR affect the heating EUI of a building, the impact of AR is not as significant as that of BH. However, the positive impact of AR is significant for retail buildings. A high AR leads to a high heating energy demand of retail buildings. As expected, SF has a significant effect on heating EUI, both in retail and residential buildings. FAR has a positive impact on the heating EUI of residential buildings. It is an important indicator of the use intensity of land, especially for residential buildings.

The results also indicated that specific variables were significant for the explanation of heating EUI of a certain building type, but not for the other building types. The possible explanations are: (1) For certain building type, the design standard limits the form and external environment of the building, which means specific parameter of this kind of building may not vary too much. Therefore, certain variable does not show significant changing trend with heating EUI. (2) The variables have relations with each other, such as BH and AR, BH and SF. We used stepwise regression approach to build the models which could exclude certain variables because of the multicollinearity. In that case, it did not mean the variable which was excluded did not have an impact on heating EUI, but its impact was not significant enough to be included.

Overall, with regard to hotels, educational buildings, and offices in severe cold regions, restrictions on building height can be appropriately relaxed to reduce their heating EUI. For retail buildings, a small shape factor can improve the heating energy efficiency. Furthermore, by increasing the street width and reducing the average BH, retail buildings, especially those at the ground floor level, can absorb more solar energy. For residential buildings, a simple slab building with low FAR is recommended.

#### 3.2.2. Climatic Parameters

The regression analysis demonstrates that climatic parameters affect the heating EUI of all types of buildings (Table 8). Considering the effect of all three climatic parameters, the R-squared value of office buildings was nearly three times that of the other types of buildings. The results of the independent samples test indicate that the regression models have statistical significance and the quantitative relations do not occur by chance.

WSP has a significant effect on all types of buildings except hospitals. The level of its impact and trends (positive or negative) vary across building types. For retail buildings, educational buildings, offices, and residential buildings, WSP inhibits heating EUI, which implies that a higher wind speed reduces the heating energy demand. The coefficient of WSP for hotels is the opposite. RH also shows different effect directions on offices and residential buildings. This mirrors the impact of temperature, whose impact varies with the type of building. As expected, the coefficients of TEMP for hotels, hospitals, and offices are negative, which indicates that the lower the temperature, the higher the heating EUI. For residential buildings, the coefficient is positive, which means that the heating EUI increases with the increase in temperature. The positive coefficients of WSP and TEMP differ from our general experience. This is possibly because the strong correlation between the independent variables causes the regression coefficients to be opposite to that of our real-world experiences [55].

### 3.3. Validation of Regression Models

**Figure 6:**
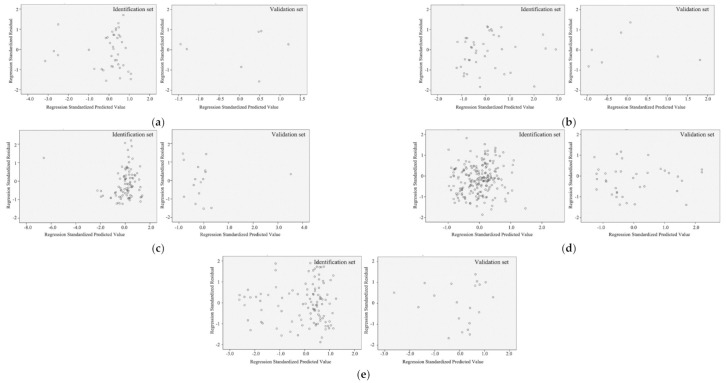
Regression standardized residual for urban morphology models: (**a**) hotel; (**b**) retail building; (**c**) educational building; (**d**) residential building; (**e**) office.

**Figure 7:**
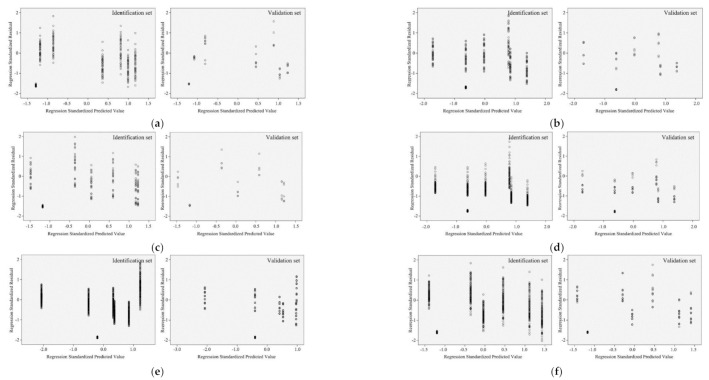
Regression standardized residual for climate models: (**a**) hotel; (**b**) retail building; (**c**) hospital; (**d**) educational building; (**e**) residential building; (**f**) office.

The residual values calculated for the identification and validation set are displayed in Figure 6 and Figure 7. Whether in the identification or validation sets, the standardized residual values were within the range of ±2, indicating that the datasets were reliable.

**Figure 8:**
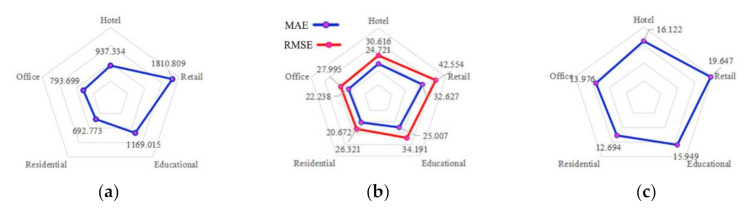
Statistical errors for urban morphology model: (**a**) MSE; (**b**) MAE and RMSE; (**c**) MAPE.

**Figure 9:**
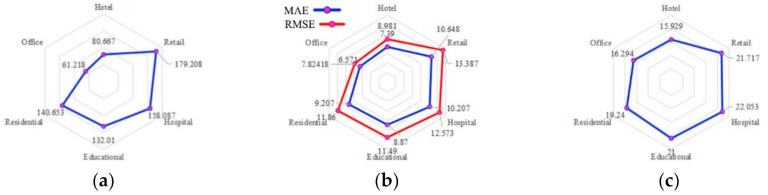
Statistical errors for climate model: (**a**) MSE; (**b**) MAE and RMSE; (**c**) MAPE.

Generally, in all cases related to urban morphology, the best correlations existed in residential buildings, followed by offices, hotels, and educational buildings, and the worst appeared in retail buildings (Figure 8). For climate-related models, the highest MSE appeared in retail buildings, while the lowest appeared in offices (Figure 9). The same conditions were valid for MAE and RMSE. As for MAPE, the best results were indicated by residential buildings, while the correlations in retail buildings were less efficient.

### 3.4. Distribution Characteristics of Heating EUI

**Figure 10:**
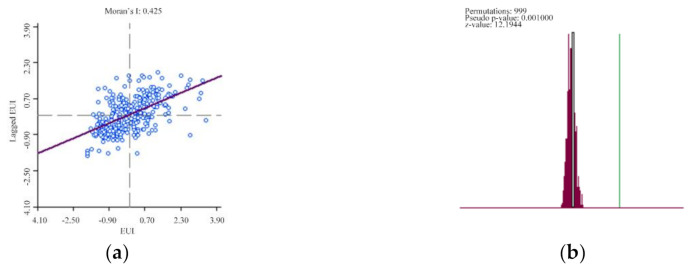
(**a**) LISA scatter plot frame of heating EUI; (**b**) the randomization test result.

Figure 10 shows the association between the heating EUI on the horizontal axis and its spatially lagged counterparts on the vertical axis. The value of Moran’s *I* is 0.425, which indicates that the heating EUI is positively spatially correlated, i.e., as the spatial distribution position gathers, the correlation becomes more significant. The results of the randomization test indicate that the spatial cluster of heating EUI is significant, as the *p*-value (0.001) is below 0.05 and the *z*-value (12.1944) is above 1.65.

**Figure 11:**
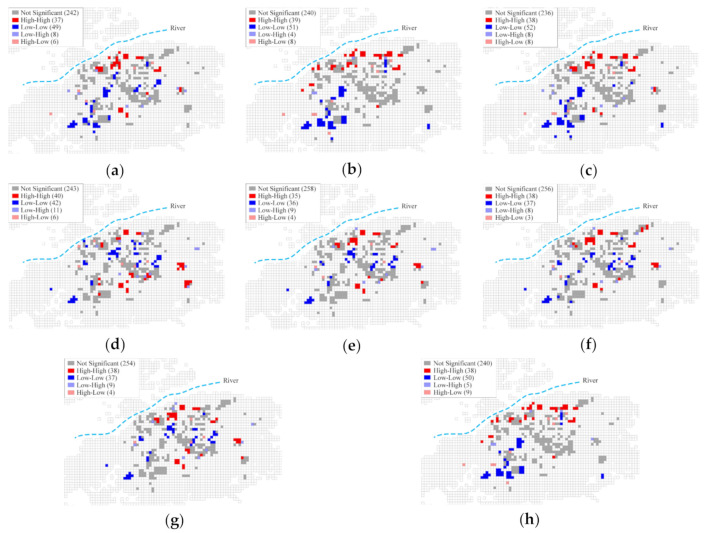
LISA cluster map of heating EUI: (**a**) annual; (**b**) January; (**c**) February; (**d**) March; (**e**) April; (**f**) October; (**g**) November; (**h**) December.

Figure 11 illustrates the LISA cluster map of annual and monthly heating EUI for all sample buildings. Consistent patterns exist in the annual and monthly maps. Based on the uniform weighting of the four neighbors distance threshold, the LISA maps show high heating EUI clusters in the north (riverside) and east of the main urban area and low heating EUI clusters in the center and southwest. This trend is more pronounced in January, February, and December, when the temperature is at its lowest.

## 4. Discussion

### 4.1. Discussion of the Results

#### 4.1.1. Impact of Urban Morphology

##### Building Height (BH)

According to the regression results, the building height has a negative correlation with heating EUI in hotels, educational buildings, and office buildings. The obtained results confirm the findings of previous research that a higher building height is more beneficial to energy savings in winter [16,40,56]. For buildings in severe cold regions, solar radiant heat is an important supplement to building heating, which they can absorb from the external environment. Existing studies find that tall buildings can better exploit the heating potential of the sun, as a larger surface area is exposed to sunlight. For a given street width, increasing building height may intensify mutual solar reflection and absorption between building surfaces [57], thereby reducing heating EUI.

##### Aspect Ratio (AR) and Floor Area Ratio (FAR)

The aspect ratio has a significant positive correlation with heating EUI in retail buildings. This research result confirmed the influence mechanism of AR on the change in building energy consumption in existing studies [58,59]. Studies in other climatic zones have shown that a higher AR leads to a smaller surface temperature change and a lower air temperature and is not conducive to the flow of air in street canyons [59,60,61,62]. With regard to severe cold regions, higher AR will store a large amount of cold air at night that cannot be easily dissipated during the day, resulting in an increase in the building cooling energy consumption. A closer building distance would also reduce the solar energy absorbed by retail buildings, especially at the ground floor level, due to the shadows from the surrounding buildings. It is worth noting that there are two main forms of retail buildings in Harbin: (1) occupying the ground floor of a commercial building, and (2) stand-alone. With regard to the retail sections at the lower floors of a building, they actually do not have a roof, but a ceiling instead. Retail sections can exchange heat with other building sections through the ceiling, which will lead to some of the “noise” in the data. However, whether it is a stand-alone building or a retail section on the lower floors of a building, the height of the retail building is usually not particularly high compared to its surrounding buildings. The impact mechanisms of AR on the heating EUI of the two forms of retail buildings are essentially the same. Therefore, in general, a high AR does not increase the heating energy efficiency in severe cold regions.

With regard to floor area ratio, many studies have discussed the relationship between FAR and building EUI, but there is still no consensus on whether a high FAR can reduce building energy consumption [57]. The regression results in this study indicate that the higher the FAR, the higher the heating EUI in residential buildings in severe cold regions. The impact mechanism of FAR is similar to AR. Usually, increasing FAR results in deep and enclosed urban canyons [59,60]. Higher FAR blocks wind flow within the urban canopy layer and stores warm air, leading to the increase in cooling energy demand in hot climate regions [63,64]. In severe cold regions, high FAR provides more shadow during the day, reduces direct solar radiation, and stores cold air, leading to the increase in heating energy demand [57,65].

##### Shape Factor (SF)

From the regression result in this study, it can be concluded that lower values of the SF correspond to lower heating EUI in both retail and residential buildings. This is consistent with the results of previous studies [66]. The shape factor represents the area of the building that is exposed to the outside air and, therefore, their potential for interacting with the climate through natural ventilation and daylight [67]. A higher shape factor implies that the external surface area of the building relative to its volume is large, leading to more heat dissipation [67]. Buildings with small volumes and complex shapes, bungalows, and low-rise buildings usually have large shape factors, which reduces their heating energy efficiency. In contrast, buildings with small shape factors, such as those with large volumes and simple shapes, multi-storey buildings, and high-rise buildings, have a lower heating EUI.

#### 4.1.2. Impact of Climate

Generally, wind speed and temperature both have a significant negative impact on building heating EUI in severe cold regions; meanwhile, relative humidity shows a positive impact. Studies have provided evidence that the urban climate environment has influences on the external surface convective heat transfer coefficient and air infiltration, and the coefficient of performance for the HVAC system, resulting in variation in building energy consumption [68,69]. Lower wind speed can reduce the heat loss from the surface and higher temperatures can raise the internal temperature of the building. Higher relative humidity is also not conducive to heat exchange on building surfaces [70].

The regression models of climatic parameters for each type of building can help to quantitatively evaluate the heating energy demand under certain climatic conditions. This is of great significance to districts and cities where central heating is the main heating method. The quantitative relationship can help heating companies or departments to adjust the heating intensity and total heating supply for different types of buildings according to changes in climatic conditions, thereby reducing waste of heating energy. For example, in China, overheating is common indoors during urban heating periods in severe cold regions, causing around 30% of excess heating [71]. Energy waste caused by overheating is particularly serious in residential buildings. Taking residential buildings as an example, the heating company can use the regression model to evaluate the actual demand for heating energy based on the WSP, TEMP, and RH forecasts provided by the meteorological department, thereby adjusting the heating scheme and reducing the heating energy supply to reduce waste.

#### 4.1.3. Impact of Location

The macroscopic spatial distribution pattern of building heating EUI in Harbin can be explained by the climatic conditions in Harbin. During winter, Harbin experiences cold winds that blow from Siberia in the north. These winds flow around the periphery of the city, making the fringes of the city colder than the center and leading to an increase in the heating EUI of the buildings located in the north and the east. The open channel space also increases the wind speed [61,62], which brings more low-temperature air from the north to the city. Therefore, a natural soft barrier of trees can be planted in the prevailing upwind direction on the edge of the urban area and along the riverside, to guide and deflect the cold winds during winter and improve the local climate [72].

### 4.2. Study Limitations and Further Research Lines

Potential inaccuracies may result from the limits of the data and the correlations between the climatic variables. Additional urban morphological factors that influence energy use also exist but are not included in this model as they cannot be easily obtained from the available sources. The prediction accuracy of the models, especially the climatic models with two or more independent variables, are affected by collinearity, but the models are still sufficiently explanatory. Studies based on other cities in severe cold regions can be conducted to further enhance these models. In addition to the impact of the urban environment, as mentioned above, the design and construction of the building itself also have a crucial impact on building energy consumption [73,74]. In this study, we controlled the variable of building envelope by some conditions in the selection of sample buildings; however, due to the large number of buildings, it was still difficult to ensure that the construction of individual buildings, such as the air tightness of buildings and the quality of the insulation installations, is exactly the same. These differences also had an impact on building energy performance [75,76], which might result in potential inaccuracies.

The study has the following three limitations, which will be addressed in future studies. (1) Limited by the actual database of building energy consumption, only five types of commercial buildings are discussed. In follow-up research, the collection and study of energy consumption data of other building types in cities in severe cold regions will be conducted. (2) Limited by the data which can be captured, the urban morphological parameters chosen in this study are not the only input variables suitable for the purpose of this study. The potential impact of other urban morphology factors will be discussed in the future. (3) The climate data used in this study are the monthly average values of the main urban area of Harbin. Collecting the climate data that matches the dynamic changes of building energy consumption is a complex and tricky task, especially for a large number of urban buildings. In a future study, we will increase the field measurement points of climate data and try to establish a more detailed one-to-one correspondence between energy consumption and climate. In addition to the three limitations mentioned above, more detailed inspection and classification of the building envelope should also be included in future studies.

## 5. Conclusions

Heating energy accounts for a significant portion of the total building energy consumption in severe cold regions and plays a decisive role in the total building energy efficiency. In this study, we developed models for urban planners that can effectively quantify the heating energy saving potential of buildings in severe cold regions. A major factor that limits decision-making in urban planning is the uncertain relationship between the impact factors and the heating energy demand of a building. The results of this study provide urban planners with an easy method of estimating the heating energy efficiency of a building, so that proper planning indicators can be set based on the function of the land.

The impact of the main environmental impact factors—morphological parameters and climatic parameters—on the potential heating EUI of buildings in severe cold regions was determined herein. We used a stepwise regression approach that only allows factors with a high level of significance to be selected. A cluster analysis revealed the spatial distribution characteristics of the heating EUI by location. Compared to simulation models based on detailed physical data and equipment, the methods used herein are based on empirical data that are self-verified and time-saving.

The impact levels of the main urban morphological factors on heating EUI were found to vary across building types. BH has a negative effect on hotels, educational buildings, and offices, and AR, SF, and FAR have positive effects on retail and residential buildings. A statistical analysis of the climatic parameters was used to determine the effects of TEMP, WSP, and RH. The results of the cluster analysis reveal that the heating EUI of buildings near the river and in the north and east of the region is high. The urban morphological limits of land areas for certain functions can be adjusted based on the significance of the influencing factors, to achieve building energy conservation. For example, the building height limits of hotels, educational buildings, and offices can be appropriately raised; the AR of retail buildings can be appropriately decreased; and low SF and low FAR can be used for residential buildings. Even so, we should note that these are not the only factors that are considered in urban morphology planning. The results of this study should act as guidelines for improving building energy efficiency while considering other urban planning factors. The quantitative relationship between climatic parameters and heating EUI provides help for heating companies and departments to evaluate the actual heating energy demand of certain types of building under certain climate conditions to avoid overheating and reduce heating energy waste. Appropriate tree-planting in the prevailing wind directions during winter and along the banks of the river can block the cold winds blowing into the city and reduce heating energy demand.

Nevertheless, despite the limitations mentioned above, the proposed method can be used to effectively and objectively define a set of environmental parameters that respond to changes in the heating energy demand of the building. The results obtained herein can not only be used to evaluate the energy efficiency of buildings, but they also clearly demonstrate the benefits of understanding the relationship between the energy demand of a building and its surroundings. Moreover, this study provides a reference for suitable parameter settings in other building energy consumption simulation models and may motivate the collection of improved data from real-time monitoring sources and statistics, especially in China.

## Figures and Tables

**Table 1:** Descriptive statistics of heating EUI.

Title	Test of Normality	Minimum (kWh/m^2^)	Maximum (kWh/m^2^)	Mean (kWh/m^2^)	Std. Deviation (kWh/m^2^)
	Sig				
Heating EUI	0.006	100.52	298.78	168.93	35.66

**Table 2:** Value range of parameters of selected buildings.

Building Type	Building Height (m)	Total Floor Area (m^2^)	Footprint Area (m^2^)	Annual Heating EUI (kWh/m^2^)	Monthly Heating EUI (kWh/m^2^)
Hotel	5.92–108.73	941.13–6036.59	172.00–4018.23	109.1–268.59	1.20–57.31
Retail building	6.00–97.00	1536.60–238,437.16	269.12–101,453.12	101.25–275.39	0.91–68.49
Hospital	3.29–57.42	810.65–76,286.13	223.51–3632.20	108.84–273.45	1.14–61.98
Educational building	3.49–57.00	503.04–85,500.63	174.10–6276.32	106.63–272.90	1.02–74.67
Residential building	11.39–111.84	158.06–45,679.20	158.06–5279.00	107.83–238.41	1.03–63.34
Office	4.00–115.25	941.61–106,209.65	50.30–5373.42	100.52–248.98	0.78–50.91

**Table 3:** Pearson correlations of heating EUI and urban environmental parameters.

Heating EUI	Urban Morphology					Climate		
	BD	AR	BH	FAR	SF	WSP	TEMP	RH
Hotel	0.347 *	−0.400 **	−0.435 **	−0.054	0.275	−0.387 **	−0.604 **	0.564 **
Retail	0.189	0.324 *	0.07	−0.236	0.365 *	−0.357 **	−0.310 **	0.347 **
Hospital	−0.156	−0.227	−0.267	0.05	−0.1	−0.394 **	−0.442 **	0.435 **
Educational	0.013	−0.270 **	−0.271 **	0.056	0.075	−0.301 **	−0.220 **	0.279 **
Residential	0.254 **	−0.160 *	−0.232 **	0.259 **	0.359 **	−0.322 **	−0.234 **	0.302 **
Office	0.105	−0.302 **	−0.355 **	−0.087	0.09	−0.620 **	−0.743 **	0.677 **

** Correlation is significant at the 0.01 level (2-tailed). * Correlation is significant at the 0.05 level (2-tailed).

**Table 4:** Pearson correlations between urban morphological parameters of hotel, retail, and educational buildings.

Title	Hotel				Retail			Educational		
	BD	AR	BH	VIF	AR	SF	VIF	AR	BH	VIF
BD	1	−0.186	−0.172	1.036	-	-	-	-	-	-
AR	−0.186	1	0.907 **	5.650	1	−0.126	1.016	1	0.853 **	3.676
BH	−0.172	0.907 **	1	5.618	-	-	-	0.853 **	1	3.676
FAR	-	-	-	-	-	-	-	-	-	-
SF	-	-	-	-	−0.126	1	1.016	-	-	-

** Correlation is significant at the 0.01 level (2-tailed).

**Table 5:** Pearson correlations between urban morphological parameters of residential and office buildings.

Title	Residential						Office		
	BD	AR	BH	FAR	SF	VIF	AR	BH	VIF
BD	1	0.049	−0.035	0.762 **	0.109	2.433	-	-	-
AR	0.049	1	0.877 **	0.122	−0.314 **	4.505	1	0.865 **	3.968
BH	−0.035	0.877 **	1	0.044	−0.392 **	4.739	0.865 **	1	3.968
FAR	0.762 **	0.122	0.044	1	0.037	2.427	-	-	-
SF	0.109	−0.314 **	−0.392 **	0.037	1	1.199	-	-	-

** Correlation is significant at the 0.01 level (2-tailed).

**Table 6:** Pearson correlations between climatic parameters.

	WSP	TEMP	RH	VIF
WSP	1	0.810 *	−0.830 *	3.300
TEMP	0.810 *	1	−0.938 **	8.475
RH	−0.830 *	−0.938 **	1	9.346

** Correlation is significant at the 0.01 level (2-tailed). * Correlation is significant at the 0.05 level (2-tailed).

**Table 7:** Stepwise regression result and *t*-test of urban morphological parameters.

Building Type	Independent Variable	Constant	α	R^2^	*t*-Test
Hotel	BH	192.037	−0.601	0.189	0.006
Retail building	AR	105.508	51.45	0.272	0.000
	SF		239.71		0.000
Educational	BH	170.211	−0.65	0.074	0.000
Residential building	FAR	99.013	9.516	0.190	0.000
	SF		217.762		0.000
Office	BH	179.642	−0.369	0.126	0.000

**Table 8:** Stepwise regression result and *t*-test of climatic parameters.

Building Type	Independent Variable	Constant	α	R^2^	*t*-Test
Hotel	WSP	−1.074	6.856	0.394	0.005
	TEMP		−1.003		0.001
Retail building	WSP	57.233	−10.281	0.127	0.003
Hospital	TEMP	23.458	−0.639	0.196	0.000
Educational building	WSP	43.923	−7.303	0.091	0.010
Residential building	WSP	0.553	−7.187	0.134	0.011
	TEMP		0.627		0.001
	RH		0.792		0.000
Office	WSP	44.302	−2.342	0.558	0.001
	TEMP		−1.060		0.001
	RH		−0.315		0.000
